# Door-to-Door Transportation Services for Reduced Mobility Population: A Descriptive Analytics of the City of Barcelona

**DOI:** 10.3390/ijerph19084536

**Published:** 2022-04-09

**Authors:** Laura Portell, Sergi Morera, Helena Ramalhinho

**Affiliations:** 1 Department of Information and Communications Technologies, Universitat Pompeu Fabra, 08018 Barcelona, Spain; 2 Institut Municipal de Persones amb Discapacitat, Barcelona City Council, 08009 Barcelona, Spain; smorera@bcn.cat; 3 Department of Economics and Business, Universitat Pompeu Fabra, 08005 Barcelona, Spain; helena.ramalhinho@upf.edu

**Keywords:** transportation, reduced mobility, disabled population, descriptive analytics, door-to-door transportation

## Abstract

A central issue in modern cities is providing inclusive transportation services for people with reduced mobility. In particular, Barcelona is offering a public door-to-door pickup transportation service complementary to the adapted regular public transport. In this work, we apply descriptive analytics to provide a detailed picture of the service by introducing and analyzing a new dataset related to this transportation service. We highlight some of the main problems of the service by processing the data associated with the users and the trips. We also suggest ideas for improving the service. Finally, we propose a trip assignment system based on priorities related to the user or trip characteristics that could improve the quality of the service.

## 1. Introduction

Globalisation has led to the development of modern cities, leading to over-populated urban areas. About two centuries ago, only 3% of the global population lived in cities. This number had increased to 54% by 2014. It is further predicted that by 2050, more than two-thirds of the worldwide population will be living in cities and urban areas [1], showing an exodus from rural life. This increase in the urban population implies changes in the socioeconomic life, cultural activities, architecture and health of the urban settlers, thereby inducing the need to adapt to the new challenges in planning city services. Importantly, people with reduced mobility should be included in the policies and decision making to create and develop these new urban cities.

For example, the city of Barcelona (Spain) comprised about 1.66 million inhabitants in 2020 [2] with a total area of 101.35 km${}^{2}$. During the last decades, Barcelona has undergone significant change due to urban planning transforming the city into a leading twenty-first-century metropolis in Europe and making it a pioneering smart city [3]. Presently, it is one of the most densely populated cities in Europe, with a high life expectancy, standing out as a city with a substantial older demographic population. In 2020, the over-65s represented 21.1% of the population of Barcelona, which is expected to rise to 35.7% by 2050 [4]. Currently, the city has 155,000 people with a legally recognized disability, representing 9% of the population of Barcelona.

Barcelona strives to improve the quality of life of its residents, especially understanding and meeting the needs of people with reduced mobility. The elderly form a part of the low-mobility population since physiological aging leads to a reduction in motor abilities, therefore causing movement difficulties [5]. To make a city more enjoyable and inclusive for Persons with Reduced Mobility (PRM), several concerns have been raised by the World Health Organization [6]. These concerns for PRM include the needs for housing, outdoor spaces, buildings, transportation, social participation, respect, social inclusion, civic participation, employment, communication, information, community support and health services. Among all the needs of these people, transportation is essential to fully participate in the economic and social life of the city [7].

One of the main goals of the Barcelona City Council and the Barcelona Metropolitan Area (AMB) is to promote mobility for all citizens, including the PRM. In this regard, a special transportation service is offered for this population, called the Special Municipal Transport Service (SMTS). The SMTS is a public door-to-door pickup transportation service offered to people with reduced mobility, which provides nonadapted and adapted taxis accessible for people with wheelchairs.

The SMTS service is offered on a first-come, first-served basis and can be booked 48 h in advance. The cost of each trip using the SMTS is priced as a single trip metro card for the user, while the institutions provide for any additional costs of service. Consequently, as the institutions have a fixed amount of money for this service, there is a financial limitation to the number of trips offered each day. One of the important challenges is that demand outnumbers the supply for the service, thereby making it, impossible to furnish all the trips demanded.

The mismatch between the demand and supply of the SMTS service therefore provides the rationale for further research to understand the nature of demand and devise ways to meet it. The research questions that this study wants to address are: How does the SMTS service work? What is the profile of the SMTS service end-users? Which factors could contribute to a better SMTS service? What kinds of trips are the most frequent? Would it be possible to have users share transports?

The aim of the study is to analyse the nature of demand for the service, understand the users as well as their needs and devise appropriate strategies to improve the service by increasing the efficiency of the trips being made (i.e., shared trips). This research aim will be fulfilled by collecting primary data and utilising descriptive analytics techniques to make meaningful findings. This research focuses on the city of Barcelona, but similar transport systems exist in other European cities such as Paris (i.e., Taxi PMR) [8] and London (i.e., Dial-a-Ride) [9], where this research can be applied.

The objectives of this study on the SMTS are threefold. The first objective is to obtain insights on the mobility of PRM by analytically describing the service offered by the SMTS. The second objective is to propose ways to improve the operational efficiency and increase the number of daily trips. The third objective is to propose a trip assignment system that prioritizes trips based on user or route characteristics.

The structure of this paper is as follows. After introducing the research subject and background information, then we will see the functioning of the SMTS and the profile and needs of the target consumers for this service. Section 2 presents the research methodology and provides the datasets and the method and tools used for the analysis. Thereafter, the results of the analysed data and a discussion of the results are synthesised in the next section. It also provides meaningful insights for improving the service. Finally, the last section draws conclusions from the study and proposes a potential future research path.

### 1.1. Special Municipal Transport Service for Persons with Reduced Mobility

Barcelona has a diverse public transport system composed of the subway, urban and intercity buses, trains, tramways, a funicular cable tramway and taxi services. Despite several modes of transportation, many stations are not easily accessible for PRM. For example, the subway system is the primary public transportation system in Barcelona. It has 8 lines and 161 stations, but 14 of them are not accessible for PRM [10]. It shows the gaps in the public transportation system, thereby impacting the mobility for PRM.

In the municipality of Barcelona, the SMTS was started in 1992 and is jointly funded and operated by the Institut Municipal de Persones amb Discapacitat (IMPD) [11], an autonomous body of the Barcelona City Council, and the AMB. It is addressed to PRM living in Barcelona by offering door-to-door service and providing them autonomy and better mobility. The SMTS is the only public door-to-door transportation service. Other private companies offer some adapted vehicles for door-to-door transportation but with a more elevated price. To use the SMTS, people must possess a white card, an accreditation processed at the Municipal Offices of Citizen Attention, which officially recognises their reduced mobility. The service is available to PRM every day of the year. The SMTS provides an integrated fleet of nonadapted and adapted taxis. Adapted taxis are the ones that have wheelchair accessibility. The popularity of the system is signified by the fact that in 2019 more than 150,000 trips were performed by the SMTS. However, in 2020, the number of trips fell to 99,215, owing to the restricted movements posed by the COVID-19 pandemic. During these two years, a total of 6591 different people used this service.

The SMTS is a complimentary public transport service for PRM. However, the cost of the service is priced as regular public transport and paid for by the institutions providing the service. This service is capped with a financial limit by the institution supporting this initiative [12]. To provide the service within the annual limit, the SMTS restricts the number of daily trips to 550. It is estimated that the daily trips available are lower than the requests made by the users, leading to a large number of requests being denied. This system is available from Sunday to Thursday from 7 a.m. to 12 p.m.. On Fridays, Saturdays, Sundays and on the eve of public holidays it is available from 7 a.m. to 2 a.m. [13].

**Figure 1:**
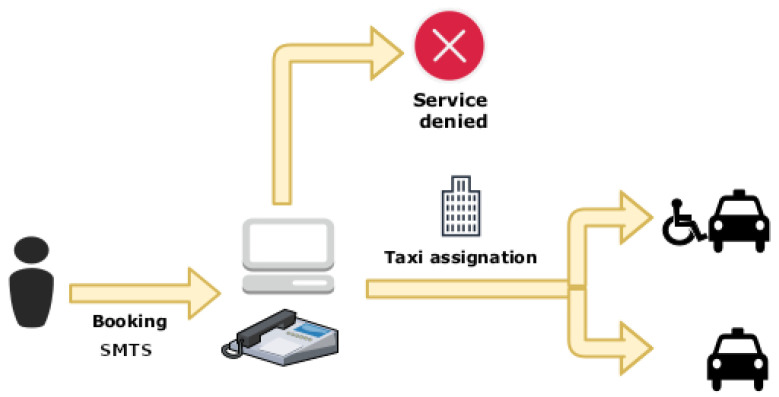
Workflow of the SMTS user request process.

The user request process for the SMTS service is illustrated in Figure 1. As given in the figure, the service request is initiated by the user by contacting the Call Centre or by filling in the form on the website at least 48 h before the trip [14]. The request is registered and processed from the Call Centre. The system thereafter evaluates the daily journey limits, available transport and disability profile of the person, and an adapted or nonadapted taxi is assigned to the accepted applications. Once all available trips are assigned, no more trips can be provided.

The eligible SMTS users are legally recognised to be disabled and, in particular, have reduced mobility. In Spain, disability recognition is conducted using a category system of 0 to 5, ranging from 0% to 100% disabled. To be legally recognised as a person with a disability, one needs to have a minimum of a 33% degree of disability and a minimum of 75% degree of disability to be classified as extreme disabled in the fifth category [15].

## 2. Materials and Methods

In this study, the data were collected from the city of Barcelona. This data describe the demographic profile of the persons with reduced mobility that are eligible to use the SMTS. The data about the trips were also analysed to understand the nature of trips availed by these users. Moreover, data relevant to insights into the current SMTS system were also collected to assess and recommend service improvements. The details of the datasets and methodology used are given in Section 2.1 and Section 2.2, respectively.

### 2.1. Data

Three different datasets were used for the analysis and are collected from various sources. The focus of the first dataset is on persons with disabilities, while the other two pertain to details about the SMTS. They are described as follows:

1. Persons with disabilities dataset: This dataset contains information about people with disabilities in the city of Barcelona, and it is annually updated by the Department of Social Affairs of the Government of Catalonia [16]. As part of a public initiative, the data are open source but preserve the anonymity of individuals. This dataset comprises information about 151,846 people with disabilities, but the relevant data extracted apply only to the persons with reduced mobility for this research. Consequently, a subset containing 41,814 PRM is used for the study. Specifically, the focus on the following information of each person is extracted: neighbourhood of residence, age and type and degree of disability.
2. SMTS datasets: Two private SMTS datasets provided by the IMPD, a department of the City Council of Barcelona, are used for this study. The first dataset contains information about the service users, whereas the second dataset describes their trips. Since the global COVID-19 emergency affected city mobility in 2020, the data cover information from 2019 to 2020.
  The SMTS user dataset contains information on 6591 people. It specifies the age, gender, type of disability, use of a wheelchair, need of a companion to travel, and the neighbourhood (refer to Table 1 for details).
  The SMTS trips dataset is composed of 254,303 trip records. Each record contains the information on the identified user, pickup start time, trip origin and destination neighbourhood, type of taxi assigned (adapted or not adapted), the trip distance (refer to Table 2 for details).
  To maintain the anonymity of the people from both datasets, the personal information that facilitates user identification, such as name, surname and ID card number, is removed. Additionally, the exact address information of the user is granulated to the neighbourhood level, that is, to the residence location of the user or the origin and destination of the trips. Explicitly, the streets are transformed into coordinates (longitude, latitude), and their corresponding neighbourhood is calculated. In the case of Barcelona, the city has 73 distinct neighbourhoods. The neighbourhood processing is performed with the GeoPandas package [17] and the shapefile (.shp) of Barcelona’s neighbourhoods [18].

**Table 1:** Examples of the structure of the SMTS user dataset. Each row in the dataset represents a different user and contains information about the user identifier, age, gender, type of disability, degree of disability, use of a wheelchair, neighbourhood residency and the total number of trips made.

User ID	Age	Gender	Type of Disability	Degree of Disability	Wheelchair	Residence Neighbourhood	Total Trips
111	27	F	Visual	75	False	Sants	26
112	83	M	Physical	45	True	Clot	2

**Table 2:** Examples of the dataset structure of the trips made with the SMTS. It contains information about trip identifier, user identifier, origin and destination neighbourhood of the trip, taxi pickup time and date, assigned taxi (adapted or nonadapted), and trip distance.

Trip ID	User ID	Origin Neighbourhood	Destination Neighbourhood	Pick up Time	Pick up Date	Taxi Assigned	Distance Trip (km)
684	111	Horta	Guinardó	7:15 p.m.	23 February 2020	adapted	2.8
395	155	Clot	Glòries	11:00 a.m.	11 November 2019	non-adapted	1.5

Other datasets are used to a minor extent to complete all the necessary data. The dataset containing a shapefile with the vectors of Barcelona’s neighbourhoods is used. This dataset makes it possible to identify each coordinate to a neighbourhood in the city. Data on average income [19], public transport stations [20], total population and age groups per neighbourhood [21] are also used.

### 2.2. Methodology

The methodology applied in this work is based on four different steps. The first step is to define the objectives by understanding the problem, defining the research questions to be solved, and identifying the data needed for this purpose. To this end, talking with experts in the subject and with users of the SMTS provided us with enormous insights. The second step is performing data collection, in which raw and heterogeneous data are collected from different sources and types (see Section 2.1). These data are needed to be integrated via data integration routines, transformed into a standard format, and saved into a database. The third step is preparing the data for analysis by fixing data quality problems. This consists of cleaning and preprocessing the data by converting the data into a structured format. For this, we needed to remove typos, categorise attributes and eliminate duplicates, outliers or irrelevant information. In addition, we converted addresses to latitude and longitude coordinates and neighbourhoods or districts for dealing with geographical data.

Once the data are ready, the last step is to analyse the data by applying descriptive analytics and visualisation techniques [22]. It consists of examining the data for answering the research questions and displaying visualisations to obtain insights from the data. In this case, it was utilised to understand the profile of the persons with reduced mobility and their transportation in the SMTS and detect the service’s main problems. Different techniques were used to this end, such as computing the correlation coefficients to analyse the relationship between variables or measuring the frequency, central tendency, dispersion of variation or position of the data. In addition, another technique was used to determine the most frequent trips between zones during a period. The frequency of these trips was measured given a time slot *t* and a day of the week *w*, for instance, Mondays between 7 a.m. and 10 a.m. For each combination k∈[1,…,w·t] of time slot and day of the week, an origin–destination ${\mathrm{OD}}_{k}$ matrix was created. In this matrix, each cell $a_{ij}$ represents the number of days on which trips exceeded a specific number of trips (known as λ threshold) between the origin zone *i* and destination *j*. In other words, the cell $a_{ij}$ corresponds to the number of days that at least λ trips are being carried out between the exact origin and destination zones and by the same day of the week and time slot.

To translate the information into a visual context, data visualisation techniques were used. The visualisations used for the time-series data are shown using line plots and heatmaps. For the geographic data, region-based visualisation and line maps were used. The former was used to determine the number of people residing in each area, and the latter observed the origin and destinations of the most frequent trips. Another technique was used to analyse the density of the people’s households per neighbourhood and draw comparisons between datasets. For this, Choropleth maps were used to gain information about the geographical points to connect data features with their corresponding spatial context. They specifically represent statistical data through shading patterns on predetermined geographical areas.

The tools used for this study are the following: Programming language *Python 3* [23] is used to gather several data processing and visualisation packages. Explicitly, *pandas* [24] and *numpy* [25] packages are used for data processing and analysis. The quantitative visualisations are completed with *seaborn* [26] and *matplotlib* [27]. Finally, the geographic data processing and visualisations are performed using *geopandas* [17] and *folium* [28] packages, respectively.

## 3. Results and Discussion

In this section, the results from the general profile of the SMTS users are reported. The trips undertaken in Barcelona with the SMTS are also assessed. The analysis of user results and trip results was carried out with the aim of detecting problems in the SMTS and finding potential improvement opportunities. To note, the SMTS should not be used regularly since other transport services are providing day-to-day service as offered by the Barcelona City Council and the AMB. Therefore, this regular service must be used to go to work or go to specific day-care centres.

Moreover, when a person with several health problems needs to receive any medical treatment, the council offers a specialist service called Salutrespon, provided by the government of Catalonia. Therefore, these two services mentioned above indicate that the use of the SMTS should be sporadic and not for regular travel.

### 3.1. Analysis of the Users

In the following section, the profile of persons with reduced mobility and their usage patterns of the SMTS are assessed.

It is estimated that Barcelona has a total of 150,000 people with disabilities. Of these, 41,814 people have reduced mobility and are eligible to use the service. During 2019 and 2020, a total of 6591 different people has used this service. Of these 6591 PRM who have used the SMTS, 69.9% have a physical disability. A physical disability can be both motor, derived from the loss of movement capacity due to various causes (malformations, accidents, brain injuries etc.), and nonmotor, derived from organic diseases (fatigue, coronary, renal, lung diseases etc.) [16]. To a lesser extent, people with intellectual disabilities (4.9%), visual disabilities (2.8%), mental disorders (1.3%) and a very few with hearing disabilities (0.5%) used the SMTS service.

#### 3.1.1. Age Range Comparison between PRM and SMTS Users

In this subsection, the age ranges of the group of PRM are compared with those of the SMTS users. Specific differences between them are assessed regarding age to determine the differences between potential candidates who could use the service and those who use it.

**Figure 2:**
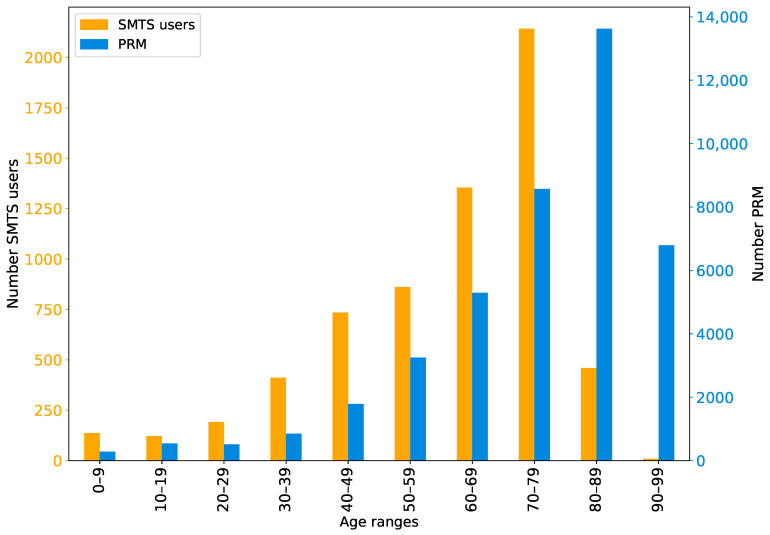
Age distribution and comparison between SMTS users and PRM. Orange bars (primary axis) represent SMTS data, while blue bars (secondary axis) represent PRM data.

As observed in Figure 2, the age distribution of SMTS users is given in orange, and the PRM in Barcelona is stated in blue. On the *Y*-axis, the scale of the total number of SMTS users is displayed on the left side, while on the right side, the total number of PRM users is given. The *X*-axis shows the different age groups.

It can be observed from the figure that the SMTS users are younger compared to the PRM (signified by the difference in orange and blue bars). As the *X*-axis represents the age of the individuals in increasing order, it indicates that the overall profile of the SMTS users has a lower age range than that of PRM. It is important to note that the mean age of SMTS users is 59.7 years, while the mean age of PRM is 74.5 years. The figure also shows that people over 90 years of age do not use the SMTS, while 16% of PRM belong to this age group. In addition, half of the PRM living in Barcelona are over 80 years old, while only 8% of SMTS users are over this age.

In summary, it can be found that users who use the SMTS service are on average younger than the potential users. One reason for the lower number of aged people using the SMTS is that these people tend to travel less and take shorter trips after retirement [29]. Consequently, older people tend to make fewer trips than younger people and may change their transport mode. Another reason could be that because older people have a shorter attention span [30], they have more problems entering the process that needs to be performed to receive this service (i.e., calling at a specific time to make a reservation so that rides are still available, waiting until the call centre is not busy and answering the call). For this reason, we believe that the reservation system should be more inclusive for older people. A third reason could be that older people do not want to go through the formalities to become a SMTS user. It should be noted that in order to use the SMTS, people must have a white card, an accreditation that officially recognises SMTS users and must be obtained through a formal process.

#### 3.1.2. Neighbourhood Differences between PRM and SMTS Users

**Figure 3:**
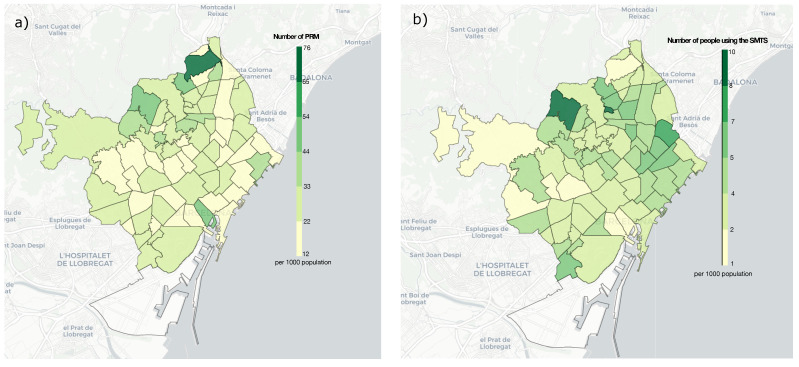
(**a**) Density of the PRM residing in each neighbourhood of the city of Barcelona (per 1000 inhabitants); (**b**) Density of the of SMTS users residing in each neighbourhood of the city of Barcelona.

This section first analyses the residence of PRM and SMTS users, followed by a comparison between them. The 73 neighbourhoods of Barcelona were considered by measuring the density of PRM and SMTS users, concerning the region’s total population expressed per 1000 inhabitants. The results are presented in Figure 3a,b through a Choropleth map and can be seen in more details in Appendix B. Before explaining each Figure in detail, it should be noted that the neighbourhood of *La Marina del Prat Vermell* (located at the bottom left of the map) has been considered a particular case due to its idiosyncrasy. That is the reason why it has not been coloured on the map. It is a small neighbourhood with a small population (see Appendix A) where residential centres and housing have been given to people with disabilities. Therefore, it has a much higher number of PRM and SMTS users. The following figures will discuss the possible factors that could influence the results, although we have not found a direct correlation between them. The factors are neighbourhood income and the number of available public transport stations (considering the underground, the *Ferrocarrils de la Generalitat de Catalunya* (FGC) and the tram) per neighbourhood. We should bear in mind that other factors could contribute to the results.

Figure 3a shows the density of PRM (per 1000 habitants) residing in the neighbourhoods of Barcelona (see Appendix B). It can be found that a higher concentration of PRM lives in the district of *Torre Baró* (76 inhabitants), followed by *Montbau* (54) and *el Barri Gòtic* (47). Assessing the income profile of the neighbourhoods, they are in the 70th, 42nd and 18th position, respectively, on the list (see Appendix A).

In Figure 3b, we can see the number of people using the SMTS (per 1000 inhabitants) in all neighbourhoods. *Montbau*, which is a neighbourhood located on the northeast side of Barcelona, presents the highest value of SMTS users (9.6), followed by *Can Peguera* (9), *la Verneda i la Pau* (6.8) and, *Sant Martí de Provençals* (6.3). It is important to mention that these neighbourhoods are near the bottom of the list regarding average income of the people living there (42nd, 65th, 59th and 54th position, respectively). Therefore, income could be an important factor in SMTS usage in low-income neighbourhoods, since people with lower incomes could take advantage of this kind of service. On the other hand, the public transport sections—taking into account the underground, the FGC and the tram—that are available in these neighbourhoods drastically change between these four top SMTS users’ neighbourhoods (see Appendix B, *No. Public Transport*). *Montbau* is in the 25th position, *Can Peguera* has no stations (it is a tiny neighbourhood, see Appendix A), *la Verneda i la Pau* is in the 48th position, and *Sant Martí de Provençals* is in the 54th. Although these are not the neighbourhoods with the best public transport, in general, they are not the worst. On the other hand, the neighbourhoods that are using the SMTS less are *el Barri Gòtic* (1.1 inhabitants), *les Tres Torres (1.6)* and *Vallvidrera, el Tibidabo i les Planes* (1.7). These neighbourhoods are listed as high-income (18th, 2nd and 10th position, respectively). Therefore, we can conclude that, in general, the neighbourhoods that make more use of the service have a lower income than the ones that use it the least.

**Figure 4:**
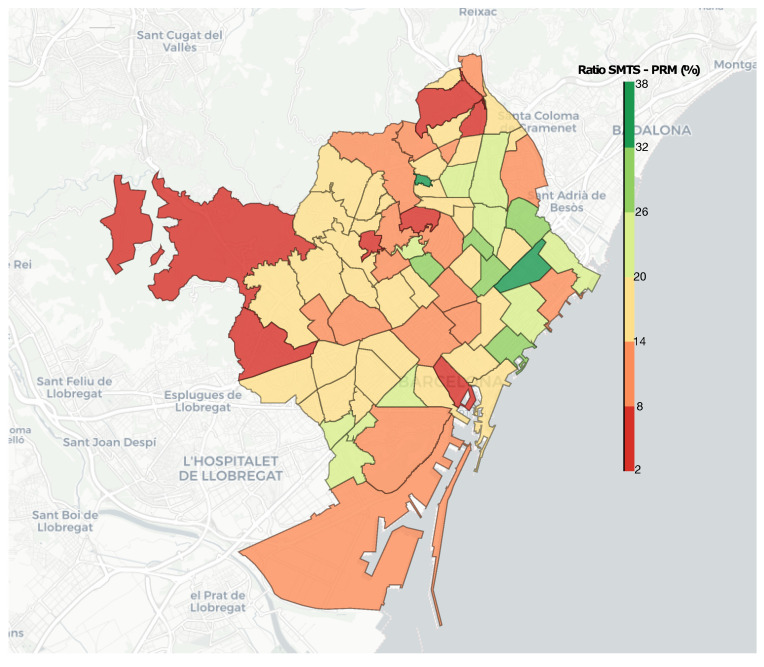
Ratio of PRM using the SMTS. The number indicates the percentage of PRM who use the SMTS in each neighbourhood. Greener colours indicate a higher percentage of PRM are using the SMTS. Redder colours indicate a lower percentage of PRM are using the SMTS.

To compare the SMTS users and the PRM, the ratio between them was calculated by dividing the number of people using the SMTS by the PRM in each neighbourhood. This approach allows us to see the percentage of PRM that use the SMTS, as shown in Figure 4 (see more details in Appendix B). In general, it can be observed that the neighbourhoods situated on the east side of Barcelona are the ones who are using the service more. In general, these neighbourhoods are the ones with lower-middle-income families. The ones located on the northwest side of Barcelona are the ones that are using the SMTS in a lower percentage of the cases. These neighbourhoods are situated in areas with higher incomes. These results show that income may be a relevant factor in explaining the use of the SMTS, while we have not found any significant associations with public transport.

Several other factors should be considered to understand the differences between neighbourhoods. The topography of Barcelona, which sits between the Mediterranean Sea and the Catalan Coastal Range, is crucial. Some neighbourhoods are situated in the hilly areas where the streets are steeper, making it more difficult for PRM to visit the closest public transport station, while others are located in flatter or plains areas. Furthermore, although we have not found a clear relationship with the amount of public transport available, it would also be interesting to analyse the waiting time to take public transport, the distance between stops, the accessibility of stations and the number of transfers needed to get from one point to another.

#### 3.1.3. Behaviour of the Users

This section analyses the behaviour of users in terms of the frequency of use of the SMTS. When examining the users’ frequency of use of the SMTS, it can be observed that the top 1% of users who make more use of the SMTS benefit from 19% of the trips. In contrast, it takes 76% of the users who use the service the least to reach the same 19% of trips, which shows disproportionality of behaviour among SMTS users. Therefore, a post hoc analysis was further conducted to investigate the behaviour of users by categorising them according to the frequency of SMTS usage. These are placed into three categories: sporadic, frequent and overactive users.

The sporadic users utilise the service once per month (or even less), while the frequent users profit from this service up to two times per week. Lastly, the overactive users are the ones who use this service excessively, more than two times per week. By the results presented in Table 3, it is worth mentioning that 12.97% of the total trips are made by 68.58% of sporadic users, indicating that these users are not the primary users of the total trips. Specifically, 37.53% of the full trips are being utilised by only 3.52% of the users, namely the overactive ones. In fact, some overactive users have undertaken more than 1000 trips. These results show the importance of categorising the trips accordingly to the behaviour of the users.

#### 3.1.4. Degree of Disability of the Users

Spain uses a category system of 0 to 5 to define the degree of disability, ranging from 0% to 100%. To be legally recognised as a person with a disability, one needs to have a minimum of a 33% degree of incapacity. To be classified as extremely disabled, and therefore part of the fifth category, one must have a minimum of a 75% degree of disability. People belonging to this category are not able to independently perform daily life’s activities such as bathing, eating and dressing [15].

To be authorised as a suitable user for the SMTS, a person must have a legally recognised disability and reduced mobility. Therefore, all the people using the SMTS should have a disability equal to or higher than 33%. It has been found from the analysed data that 56.58% of SMTS users present an equal or higher disability of 75%. It shows how people belonging to the fifth category are the ones that could take the most advantage of the SMTS as they represent the neediest. This percentage of SMTS users are also the ones representing 52% of the total trips from this system. Moreover, only 14% of the SMTS users have a disability degree less than or equal to 60%, showing how the vast majority of people using this transport have severe disabilities.

### 3.2. Analysis of Trips

The following is an analysis of the trips made in Barcelona with the SMTS, looking at the frequency of trips made, the distances involved, the most common destination points and the relationships between neighbourhoods.

#### 3.2.1. SMTS Trips Frequency

**Figure 5:**
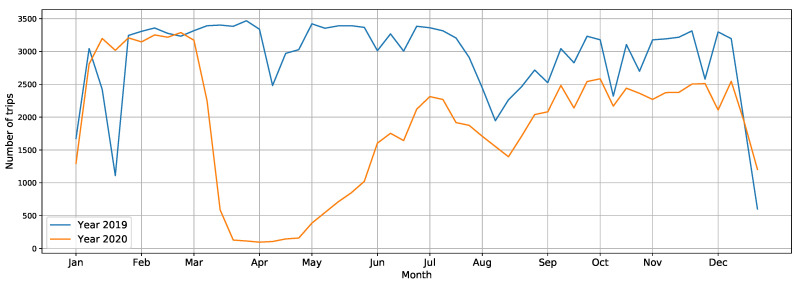
Number of weekly trips made with the SMTS during 2019 and 2020. Blue and orange line graphs represent the number of trips made during 2019 and 2020, respectively.

In this subsection, the frequency of trips throughout 2019 and 2020 are assessed. The year 2020 was an anomaly because of the disruptions of the COVID-19 pandemic. Figure 5 provides evidence of those disruptions as it shows the differences between 2019 and 2020 in the SMTS use in Barcelona. In 2019, a total of 155,088 trips were made, while in 2020, this value fell below 100,000 (99,215 trips). It shows that in 2020 only 63.98% of the trips were undertaken compared to 2019. Not only the total number of trips decreased in 2020 (as compared to the previous year), but the number of users taking advantage of the service also declined. The number of users decreased by about 10%, from 5176 in 2019 to 4777 in 2020.

In 2019, a significant downward spike can be seen throughout January. This was due to a taxi strike, showing that the SMTS could not be used. In the same year, there was a decrease in SMTS use during Easter, as people stayed at home or went on holiday for that period.

In 2020, from mid-March to June, no trips were made. During those months in Barcelona and the whole of Spain, there was a total lockdown dictated by the Spanish government to prevent the spread of COVID-19. There was a recovery in the number of trips made from June onwards, but they never returned to the number recorded for previous years.

In both these years, the vast majority of people living in Barcelona and Spain took holidays in August, which could be the reason for the decrease in SMTS use during that month. The several small dips between September and December could be explained by different holidays announced in the local calendar.

**Figure 6:**
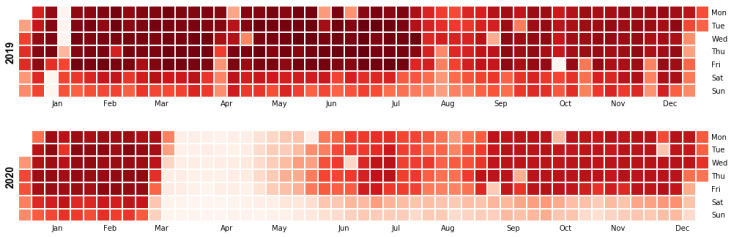
Calendar heatmap showing the number of daily trips made during 2019 and 2020.

Figure 6 shows the daily trips made during 2019 and 2020. It can be observed that the number of trips during weekends is lower than the trips made on weekdays. Therefore, as a post hoc analysis, we focused on the trips made from Monday to Friday, not considering the official public holidays in Barcelona.

#### 3.2.2. Trip Distances

In this subsection, distances of trips and differences between neighbourhoods are shown.

Barcelona has a total area of 101.35 square kilometres and is approximately 14 km long and 8 km wide. The distance of the trip between the origin point and the destination point is calculated using the geodesic distance. The geodesic distance is calculated between the origin and destination coordinates and calculated as the shortest path on the surface of an ellipsoidal model of the Earth [31]. The average distance is 3.40 km per trip if we calculate the mean distance of all trips. The average distance is also calculated for each neighbourhood by measuring the average distance between all trips departing from the same area. The neighbourhoods with the most significant difference in mean distance travelled are shown below.

The neighbourhoods where the inhabitants make the longest trips are *Vallbona* (8.73 km), *la Marina del Prat Vermell* (7.44 km) and *Baró de Viver* (6.92 km). These neighbourhoods are situated on the outskirts of the city. Therefore, if people want to travel to the city centre, they will travel a longer distance to get there. In contrast, the neighbourhoods with the shortest average trips are *el Coll* (1.39 km), *el Baix Guinardó* (2.06 km) and *la Vila de Gràcia* (2.07 km). These three neighbourhoods are located in the same geographical areas. These areas denote small streets and steep slopes. It therefore becomes difficult for users to travel to these places and induces them to use the service not only for long distances but also for shorter trips.

#### 3.2.3. Most Common Destinations

In this section, the most common destination for the SMTS trips is identified. It is found that the most common destinations are healthcare facilities, such as hospitals and primary care centres. The most popular hospitals are *Hospital del Mar*, *Hospital Universitari Sagrat Cor* and *Hospital Vall d’Hebron*. Other destinations are rehabilitation centres and, to a lesser extent, shopping centres, shops and mortuaries. In other cases, the places with no point of interest are also identified. These points correspond each time to a single user performing the displacements. Looking in more detail, this destination represents the residence of the overactive users seen in Section 3.1.3.

**Figure 7:**
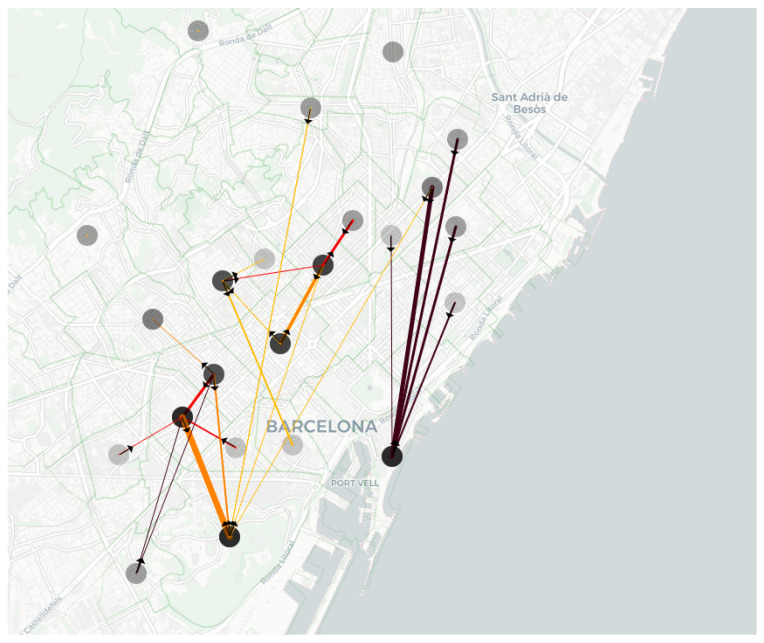
Map of Barcelona showing the most frequent journeys between neighbourhoods, showing those made more than 500 times. The dots represent the neighbourhoods of origin and destination of the trips. The colour of the lines represents the percentage of trips to health centres. The darker the colour is, the more people go to a health centre. The arrows on the lines indicate the direction of the trip. The polygons shown in very light green represent the neighbourhoods.

The main health centres in Barcelona were selected to analyse the number of trips to health centres. The trips departing from or arriving at these centres were identified for the evaluated 2-year period. It was found that 34.0% of SMTS trips were related to them. To facilitate the visualisation of the trips most frequented by users and to identify the trips to health centres, Figure 7 was created. Figure 7 shows the dots representing the neighbourhoods where most trips are made. In addition, the lines in the figure denote the neighbourhoods with the most significant number of trips among them. The thickness of the lines indicates the volume of trips between these neighbourhoods, and the colour indicates the percentage of trips made to health centres. The darker the lines are, the higher the rate of trips to health centres. Furthermore, the figure shows a point where many lines are directed, located at the bottom right of the figure. The lines to this point are very dark, indicating that a percentage higher than 75% of people go there for health reasons. Concretely, these percentages vary between 92.5% and 98.7%. This neighbourhood has the *Hospital del Mar*, a popular hospital. As can be observed, a significant number of SMTS trips are made to health centres, while they should be made with another offered service.

The lower-left part of Figure 7, shows significant movement of people. This behaviour is attributed to the existence of two important centres: *ONCE*, which is the National Organization of Spanish blind people, and *ASPACE*, a foundation dedicated to the comprehensive care of people with cerebral palsy and other neurodevelopmental pathologies. It is also observed that many people move around Barcelona’s central area of *l’Eixample*. The figure also shows how the SMTS is frequently used for trips within the same neighbourhood. Finally, it should be noted that there are health centres that are visited frequently, such as the *Hospital Vall d’Hebron*, whose users are spread throughout Barcelona and are not so focused on a limited number of neighbourhoods. For this reason, these health centres do not appear on the map.

#### 3.2.4. Most Frequent Trips between Neighbourhoods per Time Slots and Days of the Week

In this subsection, the most frequent trips between neighbourhoods and across time are analysed. Since very few trips are made during the weekends (see Figure 6), only five working days of the week are considered. From these days, five different 3-h time slots starting at 7 a.m. and ending at 10 p.m. (7 to 10 a.m., 10 a.m. to 1 p.m., and so on) are evaluated. The remaining hours are discarded because they only have a few trips (less than 1% of the trips). Therefore, the total number of combinations of time slots and days of the week is 25 (5 days × 5 time slots). For each combination *k*, the ${\mathrm{OD}}_{k}$ matrix using 2 distinct values for the threshold λ (2 and 5, see above) is created, as described in Section 2.2. This threshold filters the days on which at least λ trips were made during the same time slot and origin–destination zones.

A final OD matrix is created by summing the resulting matrices described above. In this matrix, each cell summarises the information of the filtered trips from the 25 different time slots during the evaluated 2-year period.

Aiming to reduce transportation costs, two scenarios concerning two different values of λ are considered. The first scenario is a shared taxi service by setting a threshold λ=2. Two is considered one of the threshold values, as this is the minimum number of people necessary to share a taxi service. This scenario considers that at least two or more people are doing the same origin–destination trip and the same day and time slot.

Table 4 shows the most frequent trips obtained from the ${\mathrm{OD}}_{k}$ matrices. For instance, this table highlights that the *Nova Esquerra de l’Eixample* neighbourhood is very commonplace to move around since it is a densely populated area. Moreover, it can be observed that there are short-distance trips performed within the same neighbourhood, which currently cost the same as a long-distance trip (around EUR 20 in 2020). A further study could consider the optimisation for a shared taxi service that could lead to a reduction in the costs, and at the same time increase the offerings of trips in the city. It should be remembered that the data recorded and analysed are only for the approximately 500 trips per day that are made with the service and not the actual demand, which is higher. If this service were to be implemented, all demand would be recorded, and more trips could be shared.

The second scenario lowers transportation costs by using small buses instead of taxis by setting a threshold λ=5. The umber five is considered one of the threshold values, as this is the minimum number of people needed to use a small bus cost-effectively. This case considers that five or more people can ride a bus if they follow the same origin–destination trip and the same day and time slot. Table 5 shows the most frequent trips obtained from the ${\mathrm{OD}}_{k}$ matrices for λ=5. This table shows that three possible small-bus routes can substitute for taxi services. For instance, the first two rows show one-way trips between distinct and same neighbourhoods, respectively. The last two rows show a two-way trip between *l’Antiga Esquerra de l’Eixample* and *les Corts*, starting in the afternoon and finishing in the evening. The implementation of small-bus routes requires further study that takes into account more information about the expected kind of passenger (wheelchairs, need for a companion, and so on). In this way, more optimal bus service could be offered.

### 3.3. Mobility Insights and Recommendations

In this section, insights on the improvement of the service are provided. A priority trip request service is proposed in place of the current first-come, first-served policy.

As stated in Section 1.1, one of the problems of the SMTS is that the service demand is greater than the capacity. Currently, the service is offered on a first-come, first-served basis, and it can be booked 48 h in advance by phone or website (Figure 1). The problem is that the availability of reservations only lasts for one hour due to people demanding the service precisely 48 h in advance, making it impossible to book during the remaining available hours. It would be interesting to study the variation of the 48-h booking parameter since it can be a relevant feature, although this is out of our capabilities at this time. It is recommended that better service can be achieved by changing the current policy to one based on priorities of six factors: the disability degree of the users, the service frequency, the purpose or destination of the trip, the accessibility of public transport, the time slot of the trip and the distance of the trips. Based on the overall data analysis, it is believed that the aforementioned factors should be taken into account when setting priorities.

The first factor is the degree of disability of the users. In Section 3.1.4, this factor was analysed and, in particular, for the users with a degree of disability equal to or higher than 75%. The case of severe disability seriously limits the ability to make the trips, showing that this factor is one of the most relevant to take into account and should be prioritised when assigning the trips. It is worth noting that this group of users comprises half of the trips made with the SMTS.

The second factor to be taken into account is the frequency or number of times users utilise the service. As shown in Section 3.1.3, trips are highly concentrated among certain users. When new users want to make a trip, they are generally unaware of the quick booking situation and, therefore, do not call 48 h in advance. By the time they want to make a reservation, trips are already booked. Thus, the distribution of trips should be controlled by limiting the number of trips for each person. This limitation should be implemented by considering the individual circumstances of each user. In this way, the service would not be monopolised by the same users, and better sporadic service, which is the objective of the SMTS, could be offered.

The third factor to consider is the purpose of the trip. It was found in Section 3.2.3 that more than one-fourth of the trips are to or from health centres. These should not be covered by the SMTS, as there is an alternative door-to-door service. It would therefore allow the service’s resources to be used for the other trip demands.

The fourth factor is the accessibility of the trips. Barcelona has a highly adapted public transport system; however, some stations or transfers are still not adapted. A total of 14 out of 161 stations and 3 transfer points are not adapted in the public transport system of Barcelona. Therefore, trips that allow the users to avoid nonaccessible public transport should be prioritised. In addition, accessibility in the nearby public transport stations of the origin and destination should be considered.

The fifth factor is the time at which trips are made. Rush hours can be difficult to take public transport in general, particularly if a user has reduced mobility. It is very uncomfortable and complicated to access vehicles when transport is full, and therefore, priority should be given to SMTS trips at these times. Peak hours in Barcelona are considered from 7 to 9 a.m. and from 5 to 7 p.m., as these are the hours when the people of Barcelona commute to work. In these slots, currently, 21% of SMTS trips are made.

The last factor is the distance of the trips. It is observed that many trips are made over very short distances. When analysing the accessibility of public transport needed to make these journeys, in most cases, no inconveniences were found. Therefore, another point to take into account is the distance of the trips, as analysed in Section 3.2.2. The cost of a trip is currently the same for the institutions, regardless of the distance travelled. Therefore, a trip of very short distance and duration costs the same as a long-distance trip. For this reason, it would be more convenient for users to prioritise long trips over short ones.

Now that some essential features to consider when assigning trips have been analysed, a fair trip system should be created based on the features proposed. This process is called Multi-Criteria Decision Making (MCDM) [32]. One solution would be to create a weighted priority system, which would consist of assigning weights (a priority) to each feature, giving each trip request a score. This weight function could be solved using the Simple Additive Weighting method (SAW) [33]. Another way to find the weights could be to perform scenario analysis and discuss the values with experts.

## 4. Conclusions

The main focus of this article is to provide an overview of Barcelona’s door-to-door SMTS for PRM. In addition, it aims to offer different ways to improve the efficiency of the service, making the study suitable to be replicated in other cities that offer similar services. To this end, an analysis of SMTS users and the trips made was carried out.

Analysing the user profile, we observed different factors in the user profile that influence the use of the service, such as age, income in the neighbourhood, the nearest public transport, the user’s degree of disability and the neighbourhood where they live. In addition, the trips were analysed, finding essential factors for improving the service, such as the distance, the frequency of use, the purpose of the trips and the most frequent trips. Taking into account these determinant factors on the SMTS service, the Barcelona City Council could provide a better service to the community. It was also observed that some people overuse the service, not allowing others to take advantage of it. It was also found that a large number of trips are made for reasons outside the scope of the service, such as going to health centres. Therefore, it is highly advisable to limit the number of trips and review the purpose of these trips to provide a fairer service for all users. In addition, we analysed the routes and times of the users’ trips. Several trips are being made to and from the same neighbourhoods simultaneously. For this reason, we conclude that shared vehicles, either taxis or buses, could be used, depending on the volume of trips as well as the bookings made for the service.

In this work, we studied the main factors and characteristics of the PRM transportation. However, other factors could be considered in the demand of the service, such as traffic congestion, weather, and population behaviour. In addition, we have taken as an example the service located in Barcelona; however, we strongly believe that this work can be extended to other cities such as London and Paris. We have observed that these cities have similarities with the transportation service, even if we may need to consider the idiosyncrasies of these cities to have a complete picture. We consider that these two types of limitations do not affect the conclusions and findings of this current work and can be studied in the future.

Since we have defined the priority factors related to the use of the service (i.e., the degree of disability of the users, the distance of the trips, the frequency of the service and the purpose of the trip), it would be advisable to create a future reservation management system based on priorities instead of a first-come, first-served policy. This could be accomplished by creating a function that considers all these characteristics and assigns weights to them. Two different studies can be performed to establish the weights: one by working with the experts of the SMTS service and doing a Scenario Analysis and the other one by working on the MCDM process. In addition, other features could be studied that may be of importance, such as weather, traffic, or the optimal number of hours to book in advance. Furthermore, we have seen that shared taxis or shared buses could be used to improve travel efficiency. In a future study, an optimisation system for door-to-door shared transport can be developed, which considers the different characteristics of the users. We strongly believe that a change in the service would positively affect users and the city.

## Figures and Tables

**Table 3:** Information on the number and percentage of users and trips in each category of user behaviour.

User Type	No. of Users	% of Users	No. of Trips	% of Trips
Sporadic users	4520	68.58	32,977	12.97
Frequent users	1839	27.90	125,878	49.50
Overactive users	232	3.52	95,448	37.53

**Table 4:** Most frequent trips between neighbourhoods per day of the week and time slot, ordered by the number of days with more than λ=2 trips performed in the same time period.

Day of the Week	Time Slot	Origin Neighbourhood	Destination Neighbourhood	No. of Days with More Than 2 Trips
Tue.	4–7 p.m.	*l’Antiga Esquerra de l’Eixample*	*Sant Gervasi-Galvany*	29
Fri.	1–4 p.m.	*la Nova Esquerra de l’Eixample*	*el Poble-sec*	25
Thu.	10 a.m.–1 p.m.	*la Nova Esquerra de l’Eixample*	*la Nova Esquerra de l’Eixample*	25
Mon.	4–7 p.m.	*la Nova Esquerra de l’Eixample*	*la Sagrada Família*	23
Fri.	4–7 p.m.	*el Poble-sec*	*la Nova Esquerra de l’Eixample*	22
Thu.	10 a.m.–1 p.m.	*la Dreta de l’Eixample*	*la Dreta de l’Eixample*	18
Thu.	10 a.m.–1 p.m.	*la Nova Esquerra de l’Eixample*	*el Poble-sec*	18
Mon.	1–4 p.m.	*Sant Gervasi-la Bonanova*	*les Tres Torres*	17
Mon.	7–10 p.m.	*les Tres Torres*	*Sant Gervasi-la Bonanova*	17
Tue.	10 a.m.–1 p.m.	*Sant Gervasi-Galvany*	*Sant Gervasi-Galvany*	17

**Table 5:** Most frequent trips between neighbourhoods per day of the week and time slots, ordered by the number of days with more than λ=5 trips performed in the same time period.

Day of the Week	Time Slot	Origin Neighbourhood	Destination Neighbourhood	No. of Days with More Than 5 Trips
Thu.	7–10 p.m.	*la Nova Esquerra de l’Eixample*	*l’Antiga Esquerra de l’Eixample*	7
Tue.	10 a.m.–1 p.m.	*Sant Gervasi-Galvany*	*Sant Gervasi-Galvany*	3
Thu.	4–7 p.m.	*l’Antiga Esquerra de l’Eixample*	*les Corts*	2
Thu.	7–10 p.m.	*les Corts*	*l’Antiga Esquerra de l’Eixample*	2

## Data Availability

Restrictions apply to the availability of these data. Data was obtained from the *Institut Municipal de Persones amb Discapacitat* and are available from the authors with the permission of the IMPD.

## Abbreviations

The following abbreviations are used in this manuscript:

AMB	Barcelona Metropolitan Area
IMPD	Institut Municipal de Persones amb Discapacitat
PRM	Persons with Reduced Mobility
SMTS	Special Municipal Transport Service
FGC	Ferrocarrils de la Generalitat de Catalunya
MCDM	Multi-Criteria Decision Making

## Appendix A. Neighbourhoods of Barcelona

**Table A1:** This table shows the district, number of residents, income and surface area for each of Barcelona’s 73 neighbourhoods. The population income index (RFD) represents the average level of family income available per capita of the inhabitants of the neighbourhood in relation to the Barcelona average (Index 100).

Neighbourhood Code	Neighbourhood Name	District Code	District Name	Number of Inhabitants	Population Income Index (RFD)
1	el Raval	01	Ciutat Vella	48,263	71.20
2	el Barri Gòtic	01	Ciutat Vella	21,715	106.10
3	la Barceloneta	01	Ciutat Vella	15,112	79.60
4	Sant Pere, Santa Caterina i la Ribera	01	Ciutat Vella	23,241	99.40
5	el Fort Pienc	02	Eixample	33,369	106.50
6	la Sagrada Família	02	Eixample	52,245	101.80
7	la Dreta de l’Eixample	02	Eixample	44,325	175.90
8	l’Antiga Esquerra de l’Eixample	02	Eixample	43,228	137.20
9	la Nova Esquerra de l’Eixample	02	Eixample	58,621	110.20
10	Sant Antoni	02	Eixample	38,906	104.20
11	el Poble-sec	03	Sants-Montjuïc	40,157	82.20
12	la Marina del Prat Vermell	03	Sants-Montjuïc	1227	40.00
13	la Marina de Port	03	Sants-Montjuïc	31,352	69.30
14	la Font de la Guatlla	03	Sants-Montjuïc	10,377	82.90
15	Hostafrancs	03	Sants-Montjuïc	16,203	99.00
16	la Bordeta	03	Sants-Montjuïc	19,567	79.00
17	Sants-Badal	03	Sants-Montjuïc	24,938	81.00
18	Sants	03	Sants-Montjuïc	43,763	99.00
19	les Corts	04	Les Corts	46,731	120.00
20	la Maternitat i Sant Ramon	04	Les Corts	23,968	114.20
21	Pedralbes	04	Les Corts	11,936	248.80
22	Vallvidrera, el Tibidabo i les Planes	05	Sarrià-Sant Gervasi	4710	144.10
23	Sarrià	05	Sarrià-Sant Gervasi	25,242	193.60
24	les Tres Torres	05	Sarrià-Sant Gervasi	16,617	215.80
25	Sant Gervasi-la Bonanova	05	Sarrià-Sant Gervasi	26,245	184.60
26	Sant Gervasi-Galvany	05	Sarrià-Sant Gervasi	47,915	192.10
27	el Putxet i el Farró	05	Sarrià-Sant Gervasi	30,428	144.60
28	Vallcarca i els Penitents	06	Gràcia	16,147	112.50
29	el Coll	06	Gràcia	7617	87.00
30	la Salut	06	Gràcia	13,478	109.90
31	la Vila de Gràcia	06	Gràcia	50,926	104.40
32	el Camp d’en Grassot i Gràcia Nova	06	Gràcia	35,483	105.70
33	el Baix Guinardó	07	Horta-Guinardó	26,180	92.00
34	Can Baró	07	Horta-Guinardó	9331	83.30
35	el Guinardó	07	Horta-Guinardó	37,584	79.10
36	la Font d’en Fargues	07	Horta-Guinardó	9544	92.50
37	el Carmel	07	Horta-Guinardó	32,512	54.20
38	la Teixonera	07	Horta-Guinardó	11,927	73.70
39	Sant Genís dels Agudells	07	Horta-Guinardó	7538	84.10
40	Montbau	07	Horta-Guinardó	5225	79.80
41	la Vall d’Hebron	07	Horta-Guinardó	5886	95.80
42	la Clota	07	Horta-Guinardó	709	93.50
43	Horta	07	Horta-Guinardó	28,363	79.80
44	Vilapicina i la Torre Llobeta	08	Nou Barris	26,083	63.80
45	Porta	08	Nou Barris	27,813	64.40
46	el Turó de la Peira	08	Nou Barris	16,269	51.90
47	Can Peguera	08	Nou Barris	2234	51.50
48	la Guineueta	08	Nou Barris	15,420	53.80
49	Canyelles	08	Nou Barris	6869	52.20
50	les Roquetes	08	Nou Barris	16,417	49.70
51	Verdun	08	Nou Barris	12,798	51.30
52	la Prosperitat	08	Nou Barris	27,003	56.00
53	la Trinitat Nova	08	Nou Barris	7669	48.20
54	Torre Baró	08	Nou Barris	2925	46.50
55	Ciutat Meridiana	08	Nou Barris	11,091	38.60
56	Vallbona	08	Nou Barris	1421	40.90
57	la Trinitat Vella	09	Sant Andreu	10,487	47.10
58	Baró de Viver	09	Sant Andreu	2625	68.90
59	el Bon Pastor	09	Sant Andreu	13,652	65.10
60	Sant Andreu	09	Sant Andreu	58,508	77.70
61	la Sagrera	09	Sant Andreu	29,521	77.10
62	el Congrés i els Indians	09	Sant Andreu	14,726	75.10
63	Navas	09	Sant Andreu	22,457	81.60
64	el Camp de l’Arpa del Clot	10	Sant Martí	39,262	81.70
65	el Clot	10	Sant Martí	27,069	83.60
66	el Parc i la Llacuna del Poblenou	10	Sant Martí	15,947	100.40
67	la Vila Olímpica del Poblenou	10	Sant Martí	9385	164.20
68	el Poblenou	10	Sant Martí	34,432	99.90
69	Diagonal Mar i el Front Marítim del Poblenou	10	Sant Martí	13,526	150.10
70	el Besòs i el Maresme	10	Sant Martí	25,501	60.40
71	Provençals del Poblenou	10	Sant Martí	21,523	102.30
72	Sant Martí de Provençals	10	Sant Martí	26,168	67.40
73	la Verneda i la Pau	10	Sant Martí	28,878	57.00

## Appendix B. SMTS Users and PRM per Neighbourhoods

**Table A2:** This table shows for each of the 73 neighbourhoods of Barcelona the total number of SMTS users, the density of SMTS users per 1000 inhabitants, the percentage of SMTS users among the PRM, the total number of PRM, the density of PRM per 1000 inhabitants and the number of public transport stations (metro, tramcar and FGC).

Neighbourhood Name	No. SMTS Users	Density SMTS (1000 Inhab.)	Ratio SMTS-PRM (%)	No. PRM	Density PRM (1000 Inhab.)	No. Public Transport
Baró de Viver	13	4.95	16.88	77	29.33	0
Can Baró	28	3.00	21.37	131	14.04	0
Can Peguera	20	8.95	37.74	53	23.72	0
Canyelles	37	5.39	12.25	302	43.97	3
Ciutat Meridiana	30	2.70	19.23	156	14.07	2
Diagonal Mar i el Front Marítim del Poblenou	54	3.99	11.66	463	34.23	5
Horta	88	3.10	10.35	850	29.97	6
Hostafrancs	48	2.96	17.78	270	16.66	8
Montbau	50	9.57	17.61	284	54.35	6
Navas	114	5.08	25.97	439	19.55	5
Pedralbes	29	2.43	7.49	387	32.42	6
Porta	164	5.90	22.65	724	26.03	1
Provençals del Poblenou	115	5.34	35.17	327	15.19	5
Sant Andreu	306	5.23	25.67	1192	20.37	16
Sant Antoni	153	3.93	20.68	740	19.02	13
Sant Genís dels Agudells	39	5.17	15.60	250	33.17	1
Sant Gervasi-Galvany	98	2.05	8.70	1126	23.50	10
Sant Gervasi-la Bonanova	87	3.31	15.43	564	21.49	1
Sant Martí de Provençals	164	6.27	19.98	821	31.37	6
Sant Pere, Santa Caterina i la Ribera	78	3.36	17.57	444	19.10	5
Sants	143	3.27	14.14	1011	23.10	21
Sants-Badal	83	3.33	15.09	550	22.05	4
Sarrià	102	4.04	18.15	562	22.26	5
Torre Baró	7	2.39	3.15	222	75.90	3
Vallbona	4	2.81	9.09	44	30.96	0
Vallcarca i els Penitents	65	4.03	15.19	428	26.51	7
Vallvidrera, el Tibidabo i les Planes	8	1.70	5.48	146	31.00	2
Verdun	49	3.83	14.08	348	27.19	1
Vilapicina i la Torre Llobeta	128	4.91	14.68	872	33.43	9
el Baix Guinardó	124	4.74	27.56	450	17.19	4
el Barri Gòtic	23	1.06	2.27	1015	46.74	4
el Besòs i el Maresme	99	3.88	24.87	398	15.61	12
el Bon Pastor	43	3.15	13.07	329	24.10	2
el Camp d’en Grassot i Gràcia Nova	150	4.23	19.82	757	21.33	3
el Camp de l’Arpa del Clot	174	4.43	14.29	1218	31.02	8
el Carmel	89	2.74	11.10	802	24.67	5
el Clot	145	5.36	27.67	524	19.36	3
el Coll	22	2.89	6.92	318	41.75	1
el Congrés i els Indians	80	5.43	17.13	467	31.71	5
el Fort Pienc	124	3.72	11.89	1043	31.26	10
el Guinardó	119	3.17	12.45	956	25.44	5
el Parc i la Llacuna del Poblenou	68	4.26	14.88	457	28.66	12
el Poble-sec	146	3.64	11.84	1233	30.70	12
el Poblenou	149	4.33	20.16	739	21.46	4
el Putxet i el Farró	96	3.15	18.32	524	17.22	6
el Raval	82	1.70	14.67	559	11.58	14
el Turó de la Peira	74	4.55	19.84	373	22.93	4
l’Antiga Esquerra de l’Eixample	143	3.31	17.99	795	18.39	7
la Barceloneta	49	3.24	16.61	295	19.52	1
la Bordeta	103	5.26	21.06	489	24.99	1
la Clota	3	4.23	18.75	16	22.57	0
la Dreta de l’Eixample	164	3.70	13.12	1250	28.20	49
la Font d’en Fargues	30	3.14	7.83	383	40.13	0
la Font de la Guatlla	55	5.30	21.48	256	24.67	0
la Guineueta	84	5.45	17.99	467	30.29	3
la Marina de Port	168	5.36	22.43	749	23.89	4
la Marina del Prat Vermell	26	21.19	10.53	247	201.30	23
la Maternitat i Sant Ramon	94	3.92	15.99	588	24.53	17
la Nova Esquerra de l’Eixample	192	3.28	14.31	1342	22.89	13
la Prosperitat	116	4.30	16.98	683	25.29	2
la Sagrada Família	200	3.83	13.46	1486	28.44	9
la Sagrera	129	4.37	22.79	566	19.17	6
la Salut	53	3.93	12.62	420	31.16	0
la Teixonera	33	2.77	19.76	167	14.00	2
la Trinitat Nova	22	2.87	7.69	286	37.29	8
la Trinitat Vella	29	2.77	14.65	198	18.88	5
la Vall d’Hebron	28	4.76	18.30	153	25.99	5
la Verneda i la Pau	195	6.75	30.00	650	22.51	3
la Vila Olímpica del Poblenou	38	4.05	30.65	124	13.21	5
la Vila de Gràcia	160	3.14	15.14	1057	20.76	6
les Corts	161	3.45	15.56	1035	22.15	7
les Roquetes	53	3.23	15.54	341	20.77	4
les Tres Torres	27	1.62	8.63	313	18.84	5
